# *Tropheryma whipplei* Infections, Mexico, 2019–2021

**DOI:** 10.3201/eid3105.241046

**Published:** 2025-05

**Authors:** Jesús Delgado-de la Mora, Peter Grube-Pagola, Christopher D. Paddock, Marlene DeLeon-Carnes, Alvaro C. Laga, Isaac H. Solomon, José María Remes-Troche, Jesús Javier Baquera-Heredia, Gabriel Quintero-Bustos, Juan Carlos León-Contreras, Arturo Ángeles-Ángeles, Braulio Martínez-Benítez

**Affiliations:** Weill-Cornell Medicine, New York, New York, USA (J. Delgado-de la Mora); Instituto de Investigaciones Médico Biológicas de la Universidad Veracruzana, Veracruz, Mexico (P. Grube-Pagola, J.M. Remes-Troche); Centers for Disease Control and Prevention, Atlanta, Georgia, USA (C.D. Paddock, M. DeLeon-Carnes); Brigham and Women’s Hospital, Boston, Massachusetts, USA (A.C. Laga, I.H. Solomon); Centro Médico ABC, Mexico City, Mexico (J.J. Baquera-Heredia); Laboratorio Flemming, Morelos, Mexico (G. Quintero-Bustos); Instituto Nacional de Ciencias Médicas y Nutrición Salvador Zubirán, Mexico City (J.C. León-Contreras, A. Ángeles-Ángeles, B. Martínez-Benítez).

**Keywords:** Whipple’s disease, Tropheryma whipplei, bacteria, enteric infections, Mexico

## Abstract

Whipple’s disease is rarely diagnosed in Latin America. We describe 2 patients with *Tropheryma whipplei* infection diagnosed in Mexico during 2019–2021. Diagnoses were confirmed by histopathology, electron microscopy, immunohistochemistry, and DNA amplification and sequencing analysis of the 16S rRNA gene. Clinicians should be aware of *T. whipplei* infection and associated syndromes.

Whipple’s disease (WD) is an unusual infection with protean manifestations caused by *Tropheryma whipplei*, a fastidious, slow-growing, gram-positive bacterium. Classical WD was originally described in 1907 and is characterized by polyarthritis, diarrhea, and lymphadenopathy (*1*). WD is a rare disease with a prevalence of 9.8 cases/1 million inhabitants in the United States and is most frequently identified in White men >50 years of age (*2*). WD has also been described among patients with various immunosuppressive conditions, including persons with HIV infection (*3*,*4*).

In the appropriate clinical context, WD is suspected when abundant foamy macrophages containing abundant periodic acid–Schiff stain (PAS)–positive and diastase-resistant granules are found in the lamina propria of small bowel biopsy specimens (*5*). However, WD can also be documented as a localized disease in cases of endocarditis, uveitis, isolated lymphadenopathy, encephalitis, or arthritis and negative cultures (*5*).

Classic WD has been reported extensively in western Europe, Canada, and in the United States (*6*). The disease has been described only rarely in countries in Latin America, including a probable case in Mexico (*7*–*10*). The paucity of descriptions of WD from those countries could relate to underrecognition, limited access to confirmatory diagnostic assays, or genetic characteristics of the predominant population in healthcare settings. We report 2 patients with classic WD from the states of Veracruz and Mexico City in Mexico. 

## The Study

**Figure 1:**
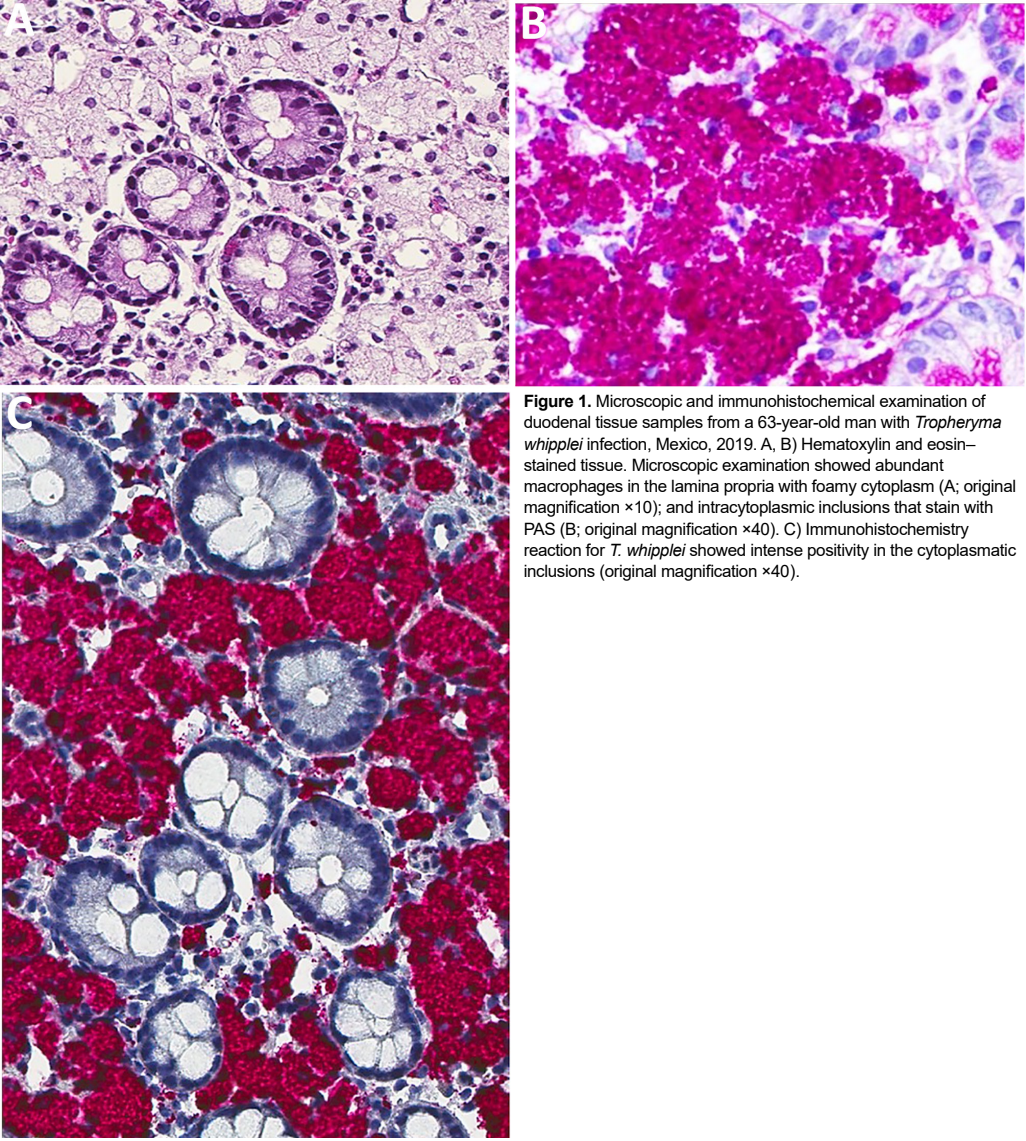
Microscopic and immunohistochemical examination of duodenal tissue samples from a 63-year-old man with *Tropheryma whipplei* infection, Mexico, 2019. A, B) Hematoxylin and eosin–stained tissue. Microscopic examination showed abundant macrophages in the lamina propria with foamy cytoplasm (A; original magnification ×10); and intracytoplasmic inclusions that stain with PAS (B; original magnification ×40). C) Immunohistochemistry reaction for *T. whipplei* showed intense positivity in the cytoplasmatic inclusions (original magnification ×40).

The first patient was a previously healthy 63-year-old man whose signs and symptoms began 2 years earlier and included cough, pneumonia, intermittent diarrhea, and weight loss of 42 kg. Results of serologic tests for celiac disease and stool bacterial cultures and ova and parasite exams were all negative. An echocardiogram revealed moderate aortic valve insufficiency and stenosis, along with severe mitral valve insufficiency, a dilated left ventricle, and mild pulmonary arterial hypertension. Small bowel endoscopy revealed multiple white nodules in the duodenum and ileum. Small bowel biopsies revealed abundant macrophages with foamy, pale, blue-gray cytoplasm in the lamina propria (Figure 1, panel A), which contained numerous globular and falciform inclusions that were strongly PAS-positive and diastase-resistant (Figure 1, panel B). Immunohistochemistry results for *T. whipplei* (*8*) were positive (Figure 1, panel C), and the PCR for gram-positive–specific 16S rRNA V8/V9 gene (*11*) produced a target 350-bp amplicon. After Sanger sequencing, *Tropheryma* spp. was identified through BLAST search (https://blast.ncbi.nlm.nih.gov). The patient initially received ceftriaxone, then received combination doxycycline and hydroxychloroquine therapy. One year after diagnosis, he had another round of duodenum and ileum biopsies, which revealed persistence of macrophages with PAS-positive inclusions; no additional studies were done. Combination therapy with doxycycline and hydroxychloroquine was reinitiated and, 2 years after those biopsies, the patient remained asymptomatic. No further endoscopic procedures were conducted.

**Figure 2:**
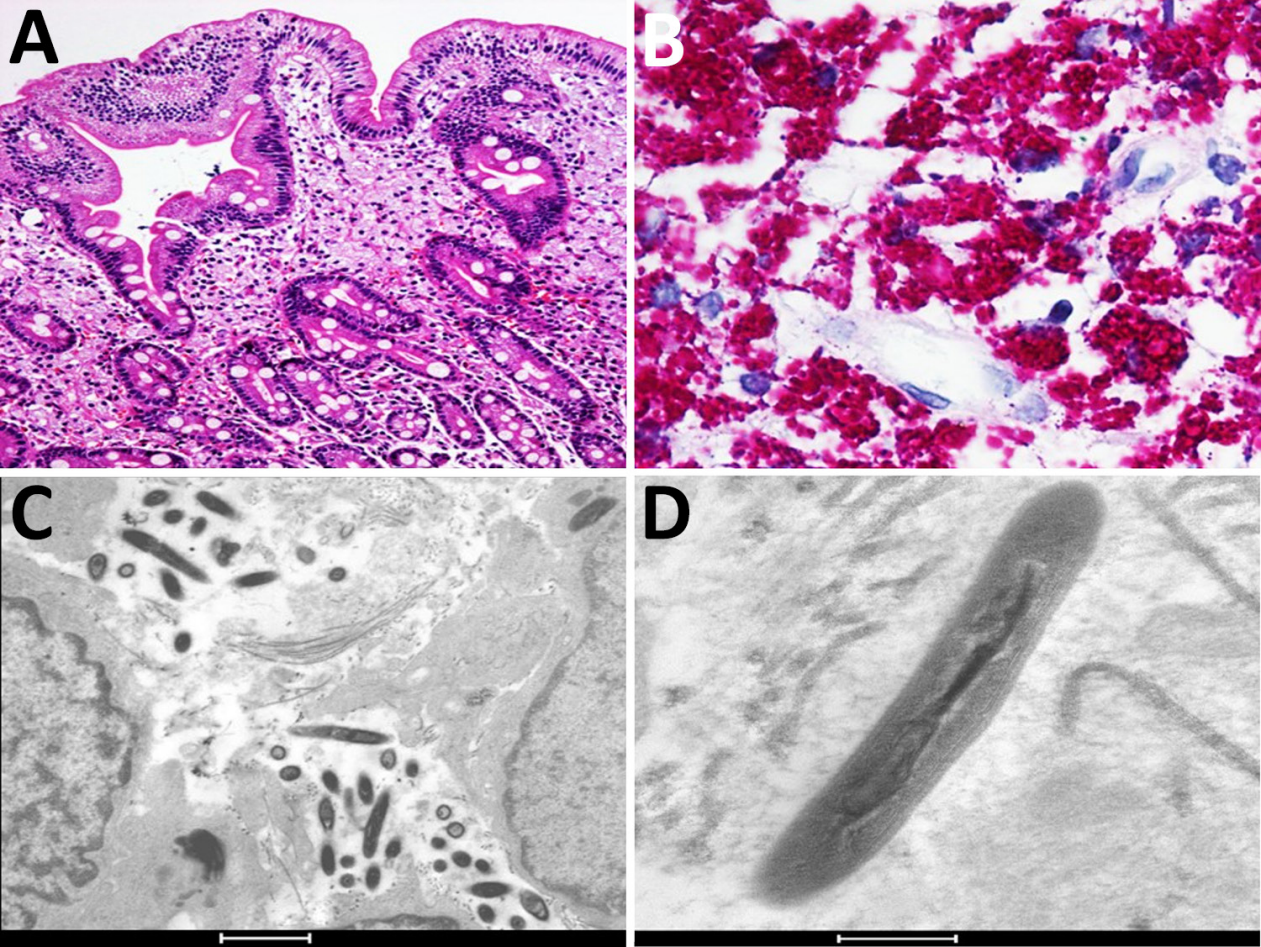
Microscopic and immunohistochemical examination of tissue samples from the ileum in a a 45-year-old man with *Tropheryma whipplei* infection, Mexico, 2021. A, B) Hematoxylin and eosin–stained ileum tissue. Microscopic examination showed abundant macrophages in the lamina propria with foamy cytoplasm (A; original magnification ×4); and intracytoplasmic inclusions that were intensely PAS-positive (B; original magnification ×40). C, D) Electron microscopy showing rod-shaped *T. whipplei* in the lamina propria of ileum (C; scale bar = 1 μm), and trilaminar plasma membranes in macrophages (D; scale bar = 200 nm).

The second patient was a 45-year-old man with well-controlled HIV infection. He was receiving raltegravir, darunavir, etravirine, and ritonavir, and his viral load was undetectable. His CD4 count was 439 cells/mm^3^. He had a history of 3 months of watery diarrhea. He received ciprofloxacin for 7 days and temporarily improved; however, his diarrhea soon returned after cessation of antibiotic therapy, and then he experienced a 4-kg weight loss. Endoscopy revealed multiple white nodular areas in the mucosa of the duodenum and ileum. Biopsy specimens from both sites revealed expansion of the lamina propria by foamy macrophages with PAS-positive, diastase-resistant inclusions (Figure 2, panels A, B). Electron microscopy performed on formalin-fixed, paraffin-embedded tissue from the small bowel (duodenum and ileum) identified intracellular and extracellular bacilli with a trilaminar plasma membrane (Figure 2, panels C, D). Molecular identification by 16S rRNA V1/V2 sequencing was performed by the Center for Advanced Molecular Diagnostics at the Brigham and Women’s Hospital (Boston, MA, USA), as previously described (*12*). A 321-bp contig was assembled and fed into the 16S RipSeq Single database (Pathogenomix, https://www.pathogenomix.com) and matched to *T. whipplei* (GenBank accession no. AF190688). We initiated doxycycline and hydroxychloroquine therapy, and his signs and symptoms resolved.

## Conclusions

We document 2 cases of WD in 2 states in Mexico. Both patients were born and raised in the country, had small bowel biopsies histologically compatible with WD, and had *T. whipplei* infection confirmed by immunohistochemical staining and molecular diagnostics.

Identification of *T. whipplei* has epidemiologic implications because of its potential for transmission between humans through saliva or feces (*5*). In addition, from a therapeutic perspective, WD generally has excellent response to antibiotic regimens; however, if such regimens are not provided, WD can result in life-threatening complications (*13*). Because classic WD frequently involves joints, it can be misdiagnosed as autoimmune rheumatologic disease, resulting in immunosuppressive therapies that paradoxically can accelerate the course of WD (*14*).

In conclusion, our findings highlight the need for more specific and widely available diagnostic tests for *T. whipplei* in Mexico and across Latin America, particularly molecular diagnostic assays, to enable characterization of *T. whipplei* in the region. Clinicians should consider *T. whipplei* infection in the differential diagnosis of patients with debilitating, otherwise unexplained, yet treatable conditions such as malabsorption syndromes and localized disease, including culture-negative endocarditis, encephalitis, uveitis, lymphadenopathies, and inflammatory spondyloarthropathies (*5*,*15*).
